# Expansivity of Fused Quartz Glass Measured Within 6 × 10^−10^ K^−1^

**DOI:** 10.1007/s10765-024-03422-3

**Published:** 2024

**Authors:** Patrick F. Egan

**Affiliations:** 1 National Institute of Standards and Technology, Gaithersburg, MD 20899, USA

**Keywords:** Dimensional metrology, Gas cells, Precision interferometry, Thermal expansion

## Abstract

A method is described to measure the thermal expansion coefficient of fused quartz glass. The measurement principle is to monitor the change in resonance frequency of a Fabry–Perot cavity as its temperature changes; the Fabry–Perot cavity is made from fused quartz glass. The standard uncertainty in the measurement was less than 0.6 (nm·m^−1^)·K^−1^, or 0.15 %. The limit on performance is arguably uncertainty in the reflection phase-shift temperature dependence, because neither thermooptic nor thermal expansion coefficients of thin-film coatings are reliably known. However, several other uncertainty contributors are at the same level of magnitude, and so any improvement in performance would entail significant effort. Furthermore, measurements of three different samples revealed that material inhomogeneity leads to differences in the effective thermal expansion coefficient of fused quartz; inhomogeneity in thermal expansion among samples is 24 times larger than the measurement uncertainty in a single sample.

## 1 Introduction and Motivation

An experimental effort is underway to measure the refractivity of helium gas at the level of 1 part in 10^6^ . The motivation is that a precision measurement of helium refractivity at known temperature allows a realization of the pascal, in what is sometimes called the optical pressure scale [1]. The underlying principle is the ideal gas law, which defines pressure in terms of density and thermodynamic temperature, together with the molar gas constant (a fixed value). The interest in helium (refractivity) is because the Lorentz–Lorenz equation provides a direct link between refractivity and density via the polarizability. Polarizability is a fundamental property of a single atom, and for helium, it can be calculated [2] well within 1 part in 10^6^. Consequently, the realization of optical pressure provides a well-understood physical system, in which all input parameters are known without reference to an ancillary measurement of pressure. The ultimate accuracy of this new scale will approach how well the thermodynamic temperature of the helium gas is known.

To measure refractivity, an approach based on gas cells is being pursued. The scheme closely follows the effort of Egan et al. [3] to determine the Boltzmann constant, with the working principle of making window pathlength error common-mode in measurements of refractivity performed in cells of different lengths. Two design tweaks have improved the concept of Ref. [3]: the cells and windows have been made in fused quartz glass, and the length of the long cell is 0.5 m. These two design tweaks should achieve (uncorrected) window pathlength error at 90 parts in 10^6^ for helium refractivity, and common-mode cancelation techniques are expected to reduce this by more than an order of magnitude.

At these expected levels of accuracy, uncertainty in gas temperature will become the dominant contribution to uncertainty to the realization of optical pressure. Therefore, the helium measurements will be performed with direct thermometry comparisons to the fixed points of water and gallium, at 0.01 °C and 29.76 °C, respectively. However, cell length is determined by coordinate measurement, which is performed at the 20 °C reference temperature of dimensional metrology. So, the requirement is to scale the dimensional measurements made at 20 °C to the refractometry operating temperature of 0.01 °C, while introducing error no larger than 100 nm on cell length. An error of 100 nm on the 0.5 m cell length would correspond to fractional error of 0.2 parts in 10^6^ in the refractivity measurement. The coefficient of thermal expansion (CTE) of fused quartz glass is approximately 0.4 (μm·m^−1^)·K^−1^. Therefore, scaling the 0.5 m cell length from 20 °C to 0.01 °C without introducing length errors greater than 100 nm requires knowledge of CTE within 10 (nm·m^−1^)·K^−1^, or 2.5 %.

Vitreous silica was a standard reference material for thermal expansion for many decades [4]. However, the glass exhibits variations in its CTE, which have been attributed to recipe, process preparation, and thermal history [4-7]. The gas cell assemblies for the newly designed refractometer are made with tubes of Type-I electric-fusion quartz glass. However, the thick wall tubes were made of a lamination of an inner and outer tube, so one can not be certain of the thermal history of the material. The windows on the cell assemblies are Type-III synthetic-fused silica glass (colloquially known as UV-grade fused silica). Wang et al. [8] show variations in $\int \alpha (T)\;dT\equiv \frac{\Delta L}{L}$ less than 1 μm·m^−1^ between Type-I and Type-III glasses across 0.01 °C to 29.76 °C, but material mismatch might cause a small end effect. Another more important potential end effect is that the tubes are bonded into end-blocks with a potting compound. The potting compound is a paste of silica powder, mixed with monoaluminum phosphate solution; the tubes were potted into the end-blocks with a slip fit of 50 μm, and fired to 300 °C for 24 h. The potting compound has a generic mean CTE specification of 0.59 × 10^−6^ K^−1^ . Based on these circumstances and a potential mismatch in expansion, it was considered critical to measure the CTE of a “mock cell” sample, upon which to base the estimate of the effective CTE of the cell assembly. (The supplemental material has photographs of the cells, cavities, and tubes.)

Before describing the methods and results, it should be noted that the present work reports all results on the International Temperature Scale of 1990 (ITS-90). The convention below follows the status quo [9], and no distinction is made between equations that originate in true thermodynamic temperature T versus measurements that access the quantity $T_{90}$. Current knowledge of thermodynamic temperature [10] shows $T-T_{90}$ to vary by about 6 mK across the (263<T<313)K temperature range of interest. For this work, temperature-scale error is about 12 times larger than any other contributor to uncertainty in the specimen temperature. This error probably has no practical consequence, but it may be removed by reanalyzing the data given in the supplemental material. (The supplemental material lists measured temperature on ITS-90. To derive a thermodynamic thermal expansion coefficent, the measured temperature would need to be converted to thermodynamic temperature using a correction function such as Ref. [10].)

## 2 Measurement Approach

The situation is that the lengths of the cells are measured by coordinate-measuring machine at a reference temperature $T_{\text{ref}}=293.15\;K$. The actual cell length at the refractometry working temperature $T_{\text{TPW}}=273.16\;K$ requires that the measured length $L_{\text{ref}}$ (i.e., $L_{\text{CMM}}$) is corrected:

(1) $$L=L_{\text{ref}}\left[1+\int_{T_{\text{ref}}}^{T_{\text{TPW}}}\alpha_{\text{cell}}(T)\;dT\right]$$

for the thermal expansion coefficient $\alpha_{\text{cell}}(T)$, which is the unknown.

**Fig. 1:**
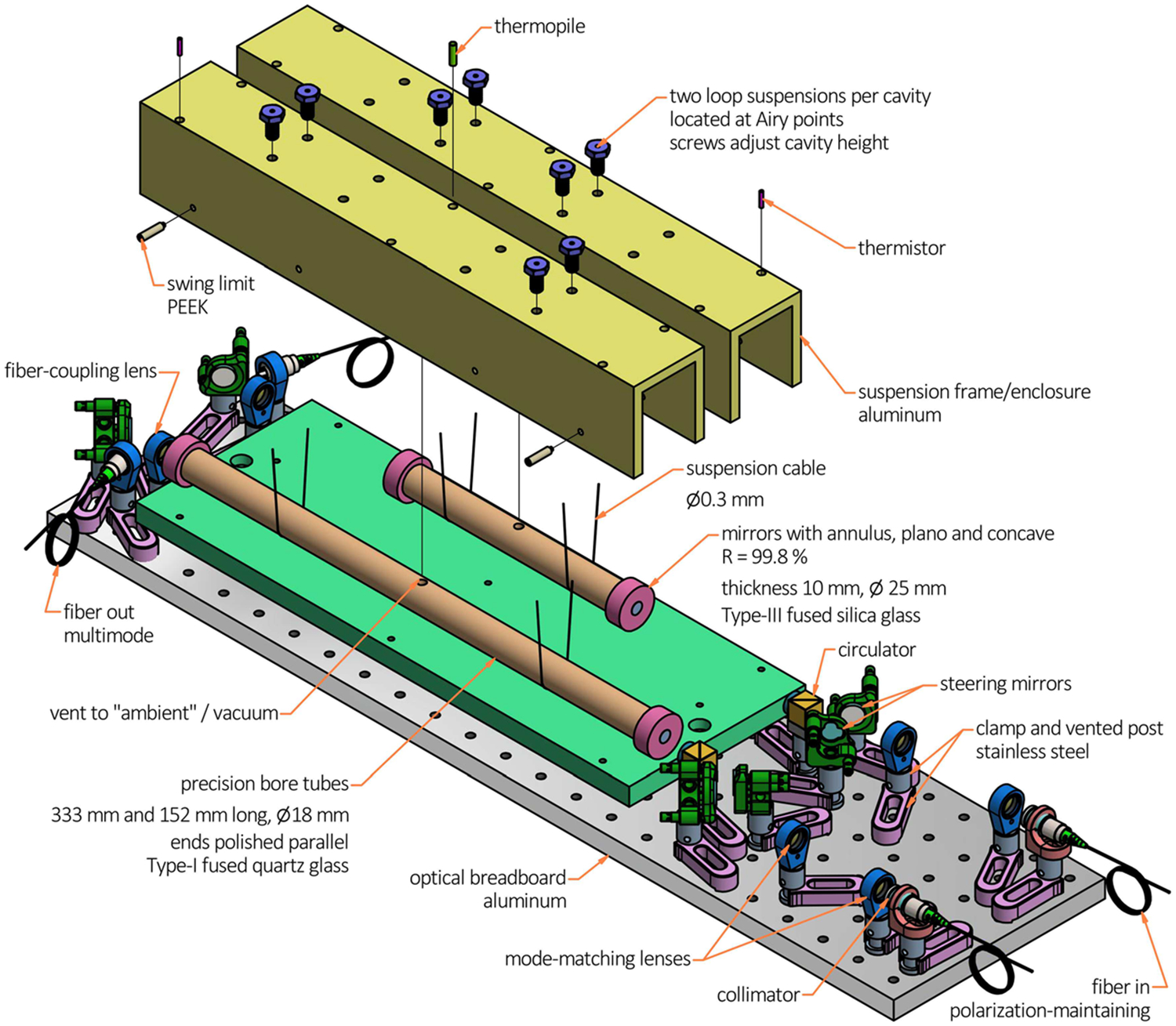
Setup for the thermal expansion measurement. Two Fabry–Perot cavities were formed out of excess (cell) tubing material, and suspended side by side. The sketched assembly was placed inside an inner shell and vacuum chamber, which was submerged in a water bath. Temperature of the water bath was varied between −10 °C and 40 °C

The mock cell samples used to infer $\alpha_{\text{cell}}(T)$ are Fabry–Perot (FP) cavities, which have spacers made from tubes of the same material batch as used in the cell assemblies; that is, Type-I fused quartz glass. The FP cavities were formed by polishing the end faces of the tubes parallel and silicate-bonding mirrors to each end. The measurement principle [11] is to track changes in the resonance frequency of each cavity as a function of temperature, relative to the reference frequency of an iodine-stabilized laser. As such, the metrology scheme closely resembles that of Ref. [12], comprising a pair of tunable HeNe lasers, dither locked to the resonance peaks of each cavity, and some frequency metrology. The metrology assembly is sketched in Fig. 1 and is entirely high-vacuum compatible. The two FP cavities were placed side by side in their own suspension frame enclosure and were suspended by 0.3 mm diameter cable at their Airy points. The temperature of each aluminum suspension enclosure was measured with a thermistor embedded in a thermowell. These two thermistors had been calibrated on ITS-90 relative to a standard platinum resistance thermometer. The apparatus of Fig. 1 was placed in a vacuum chamber and submerged in a 150 L stirred water bath; fiber inputs and outputs were fed through the water and chamber. This paragraph completes the basic description of the method—the change in resonance frequency of the tube cavities was measured as a function of temperature—further details are left to Ref. [12] and outlined in App. 1.

The (approximate) resonance frequency of a FP cavity at vacuum, $\nu \approx \frac{mc}{2L}$, depends on cavity length L and the integer mode number m; the speed of light in vacuum c is a constant. Consequently, changes in the length of the cavity may be inferred by measuring the change in resonant frequency via the difference equation $\frac{dL}{L}=-\frac{d\nu }{\nu }$, while updating dν for the change in mode number Δm, caused by the changes in cavity temperature. (In these measurements, $\frac{d\nu }{dT}\approx 180\;\text{MHz}\cdot K^{-1}$. For the 50 K change in the long cavity temperature, Δm=30.) From the inferred specimen (cavity) length as a function of temperature L(T), one may deduce the instantaneous CTE $\alpha (T)=\frac{1}{L_{\text{ref}}}\frac{dL}{dT}$. Since this experiment deals with specimens of different lengths, it is expedient to fit fractional length $\frac{L(T)}{L_{\text{ref}}}=a_{0}+\sum_{i=1}^{3}\frac{a_{i}}{i}{\left(T-T_{\text{ref}}\right)}^{i}$ about the reference temperature $T_{\text{ref}}=293.15\;K$. The resulting fit coefficients then describe instantaneous CTE as a functional approximation:

(2) $$\alpha (T)=\sum_{i=1}^{3}a_{i}{\left(T-T_{\text{ref}}\right)}^{i-1}.$$

This simplified treatment has ignored the temperature-dependent effects in diffraction and mirror phase shift on reflection—the latter has a temperature dependence which is non-negligible, and will be discussed in the uncertainty section below.

### 2.1 Results for Two Sets of CTE Measurements

Two sets of CTE measurements were performed. The first set cycled the cavities ${\text{FP}}_{152}^{\text{Type-}I}$ and ${\text{FP}}_{333}^{\text{Type-}I}$ side by side, and the second set cycled ${\text{FP}}_{152}^{\text{potted}}$ and ${\text{FP}}_{333}^{\text{Type-}I}$ side by side. For the second set, the 152 mm cavity had been potted with slip-fit tubes, while the 333 mm cavity remained unchanged between the two sets of measurements. This second set of measurements is the basis for the estimate $\alpha_{\text{cell}}(T)$.

**Fig. 2:**
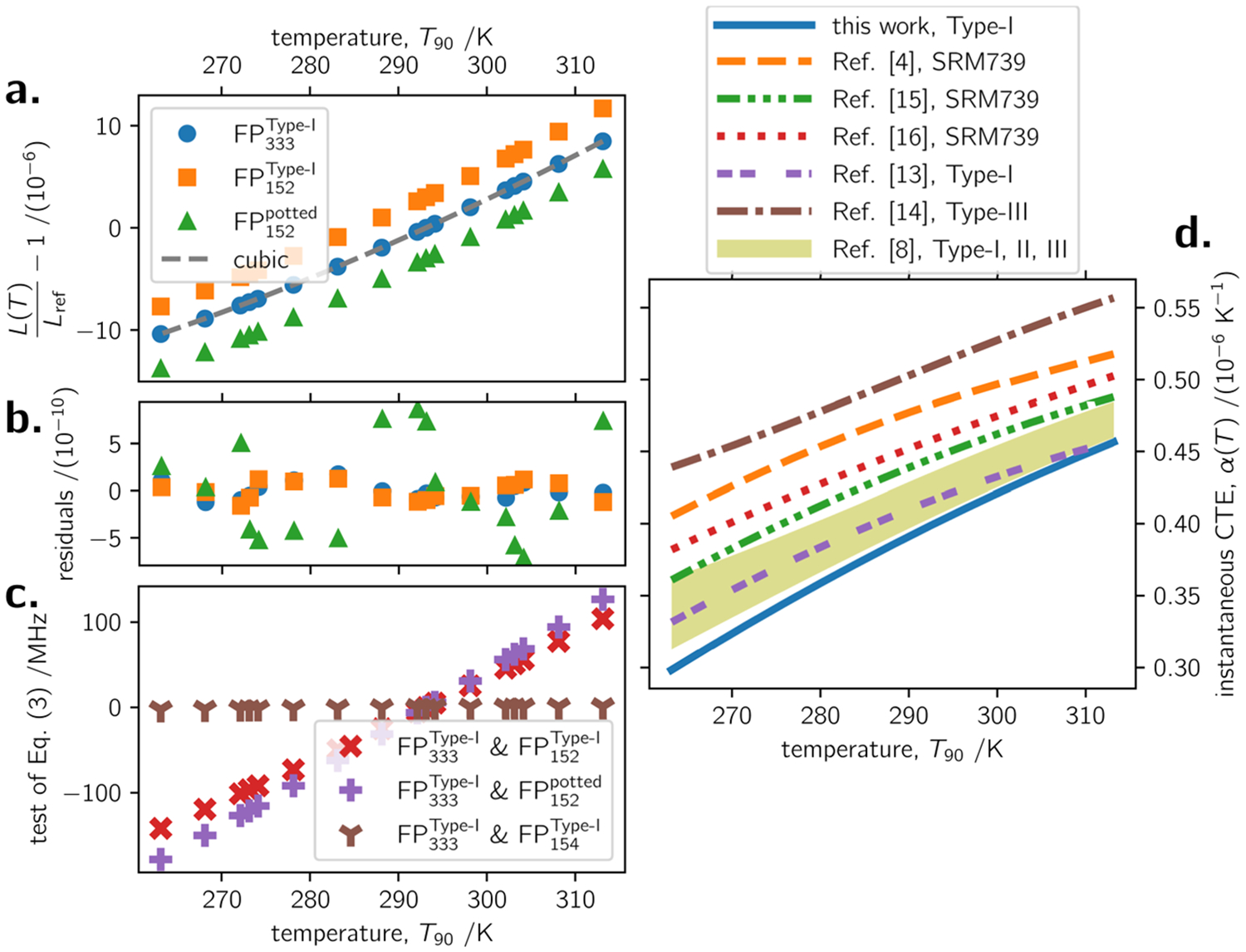
(a) Change in fractional cavity length as a function of temperature for Type-I fused quartz glass ${\text{FP}}_{333}^{\text{Type-}I}$ and ${\text{FP}}_{152}^{\text{Type-}I}$, and the potting-compound-modified ${\text{FP}}_{152}^{\text{potted}}$. The plots are offset by 3 × 10^−6^ in the ordinate for clarity. (b) Residuals from the fit: the $\frac{L(T)}{L_{\text{ref}}}$ dataset for each FP cavity has its own fit coefficients. (c) Anomalous change in the intercavity beat frequency, defined as (3). The slope of the trends is proportional to inhomogeneity $\Delta \alpha \approx \frac{1}{\nu }\frac{\Delta f_{\text{beat}}}{\Delta T}$ between the specimens being compared. (d) The deduced thermal expansion for Type-I fused quartz glass, as (2) in this work. Literature measurements are also shown

In Fig. 2(a), both sets of measurements are plotted as fractional change in cavity length as a function of temperature. For each cavity, the $\frac{L(T)}{L_{\text{ref}}}$ dataset was regressed to a cubic function, and produced coefficients specific to each cavity. The question of how much these sets of coefficients differed from one another is best answered by the diagnostic described in the next paragraph. Figure 2(b) shows residuals from the fits to fractional length, where the ordinate corresponds to $\frac{L(T)}{L_{\text{ref}}}-[a_{0}+\sum_{i=1}^{3}\frac{a_{i}}{i}{\left(T-T_{\text{ref}}\right)}^{i}]$. The plot shows residuals on the specimens ${\text{FP}}_{152}^{\text{Type-}I}$ and ${\text{FP}}_{333}^{\text{Type-}I}$ from the first measurement set, and specimen ${\text{FP}}_{152}^{\text{potted}}$ from the second measurement set. (For clarity in Fig. 2, the second measurement set for ${\text{FP}}_{333}^{\text{Type-}I}$ is not shown. Using the metric ∫α(T)dT, the two measurements of ${\text{FP}}_{333}^{\text{Type-}I}$ reproduce within 0.7 nm·m^−1^ across the 50 K range.) The root-mean-square error from the fits of the first measurement set is 82 pm·m^−1^ for ${\text{FP}}_{333}^{\text{Type-}I}$ and 94 pm·m^−1^ for ${\text{FP}}_{152}^{\text{Type-}I}$. The residuals for ${\text{FP}}_{152}^{\text{potted}}$ are 5.5 times larger than ${\text{FP}}_{152}^{\text{Type-}I}$, which is most likely an increase in cavity instability caused by the potting compound, because the ${\text{FP}}_{333}^{\text{Type-}I}$ residuals for the second set of measurements are within 15 % of the first set—it is no surprise that a potting compound increases instability compared to bulk material. The fit residuals for long and short cavities show obvious correlation, and the shape of the residuals persists in both sets of measurements, which suggests a systematic error affecting the length of both cavities during the measurement cycle. The most likely cause for the correlated residuals is error in thermometry, on the order of $\delta T=\frac{1}{\alpha }\frac{\delta L}{L}\approx 0.3\;\text{mK}$.

A key experimental diagnostic is the intercavity beat: that is, the < 1 GHz difference in frequency between the two lasers locked to a resonance frequency in each cavity. For cavities of identical material properties experiencing identical temperature changes, the intercavity beat would remain constant as a function of temperature. It stated more precisely,

(3) $$f_{f}-\frac{f_{\text{ref}}+\left(\frac{\Delta m_{33}\cdot c}{2L_{33}}-\frac{\Delta m_{15}\cdot c}{2L_{15}}\right)}{1+\int_{T_{\text{ref}}}^{T_{f}}\alpha (T)\;dT}\overset{?}{=}0.$$

Or, the change in intercavity beat measured at two different temperatures $T_{\text{ref}}<T_{f}$—when adjusted for changes in mode order of the respective cavities and scaled for their increasing length—should be zero, if the cavities have the same coefficient of thermal expansion, experience the same temperature change, and have no end effect. Figure 2(c) belies these assumptions and the potential equality of (3), demonstrating a linear trend of about 4.9 MHz·K^−1^ in the case of ${\text{FP}}_{152}^{\text{Type-}I}$ and ${\text{FP}}_{333}^{\text{Type-}I}$ dataset. A temperature-related explanation for the trend is implausible—it would require an undetected change in gradient between the cavities of 1.25 K, which is more than three orders of magnitude larger than what was measured. Two considered end effects are a mismatch in CTE between the tube and mirror substrates, and a temperature dependence on cavity round-trip phase. Combined, these two end effects are about a factor 30 too small to explain the changing intercavity beat. Therefore, the most likely explanation behind the trend of Fig. 2(c) is inhomogeneity in the CTE of fused quartz glass among different tubes, corresponding to $\Delta \alpha \approx \frac{1}{\nu }\frac{\Delta f_{\text{beat}}}{\Delta T}$, or 1.1 × 10^−8^ K^−1^. The magnitude of this inhomogeneity—2.5 %—is somewhat surprising but should be placed in context of a thorough work by Jacobs et al. [13], which compared thirteen different samples of fused quartz glass, core drilled from distant locations in four separate ingots (glass melts). These authors showed a location-dependent gradient in α(T) across each ingot, and the maximum variation in α(T) among three of the four melts was 5 × 10^−9^ K^−1^. They chose to “remove” data from the fourth melt because “a different grade of crystalline quartz [was] used to yield a reduced bubble content.” Nevertheless, this fourth melt would still be classified as a Type-I fused quartz glass, and if it is included in the data analysis, Jacobs et al. [13] showed variations in α(T) of up to 1.3 × 10^−8^ K^−1^ among the thirteen samples from four separate melts. (See, in particular, Fig. 13 of Ref. [13] by Jacobs et al.) The CTE inhomogeneity of the present result in Fig. 2(c) is therefore not too surprising. However, the present result for absolute CTE in both specimens, discussed next, also requires mediation. [For purposes of the uncertainty evaluation which follows in the next section, a third specimen ${\text{FP}}_{154}^{\text{Type-}I}$ of the same material was constructed. It is shown in Fig. 2(c) that ${\text{FP}}_{154}^{\text{Type-}I}$ has close agreement in α(T) to the specimen ${\text{FP}}_{333}^{\text{Type-}I}$. The relevance of this result is discussed more in the uncertainty section.]

In Fig. 2(d), it is plotted α(T) deduced from these measurements, expressed by (2). The present measurements are compared with the existing literature [4, 8, 13-16] in this temperature range. There are at least four notable aspects to the literature, interpreted through the following anecdotal commentary:

- The first is evident in Fig. 2(d) as the difference between the trends “Ref. [4], SRM 739,” “Ref. [15], SRM 739,” and “Ref. [16], SRM 739.” Okaji and coworkers have consistently reported [8, 16] a bias of 2 × 10^−8^ K^−1^ to 3 × 10^−8^ K^−1^ between their measurements of SRM 739 versus what was originally reported by Hahn and Kirby [4]. This bias is within the mutual standard uncertainties for α(T), which was 3 × 10^−8^ K^−1^ for Hahn and Kirby and 2 × 10^−8^ K^−1^ for Okaji and coworkers. Drotning [15], whose measurements of α(T) in SRM 739 are also lower than Hahn and Kirby, does not make a clear uncertainty statement, but says that the 2.7 × 10^−8^ K^−1^ standard deviation on their measurements was “near the estimated device uncertainty.” The anecdotal conclusion of this first point is that the measurement of Hahn and Kirby [4] overestimates α(T) for SRM 739.
- The second notable feature of Fig. 2(d) is the offset between data for SRM 739 compared to the shaded area plot of “Ref. [8], Type-I, II, III” which covers the range of fit data reported for all types of vitreous silica glass measured by Wang et al. [8]. This offset is notable because SRM 739 is nominally a Type-I fused quartz glass, and yet its thermal expansion coefficient (i.e., Ref. [16]) is outside the range of silica glasses investigated by Wanget et al. and measured with the same apparatus. The origin of this offset is not clear, but Wang et al. suggest that it may be related to the manufacturing process. (A subsequent article by the same Okaji group [17] extends the valid temperature range, and its findings are consistent with Refs. [8, 16].) The anecdotal conclusion of this second point is that the recipe, process, or preparation of SRM 739 produces a Type-I fused quartz glass with α(T) relatively higher than other Type-I glasses.
- The third notable aspect of Fig. 2(d) is the 9 × 10^−8^ K^−1^ offset between Ref. [14] and Ref. [8] for Type-III fused silica glass; when these two Groups performed a bilateral comparison [18] with the same SRM 739 specimen, they had agreement within 2 × 10^−8^ K^−1^ and claimed standard uncertainties on the order of 0.9 × 10^−8^ K^−1^. The anecdotal conclusion of this third point is that the same process or preparation (of Type-III fused silica glass) may produce variability as large as the range of all data in Fig. 2(d).
- Finally, the work of Jacobs et al. [13] should be mentioned. (Ref. [13] does not list fit coefficients, but a plot digitizer [19] was used to interpolate their Fig. 1.) Jacobs and coworkers pioneered the FP cavity-based approach to CTE measurement for at least three decades [11, 13, 20-22]. Arguably, Ref. [13] was the pinnacle in their body of work; in Ref. [13] they demonstrate reproducibility at the level of 1 × 10^−9^ K^−1^ and make a thermometry-limited uncertainty claim of 4.5 × 10^−9^ K^−1^ in the measurement of a Type-I fused quartz glass. The anecdotal conclusion of this fourth point is that the hitherto most accurate claim on α(T) for a Type-I fused quartz glass is notably lower than older data in Fig. 2(d).
- Parenthetically, also mentioned are two older sets of measurements which are not plotted in Fig. 2(d). Berthold and Jacobs [20] do not give fit coefficients, but their α(T) plot for a Type-III specimen appears in good agreement with the Type-III measurements of Birch [14]; however, despite claimed precision of 1 × 10^−9^ K^−1^, one of Berthold and Jacobs’ three specimens differed by 4 × 10^−8^ K^−1^ from the others. The second older measurement for a Type-III specimen was by Bennett [23] and is also in good agreement with Birch [14]; however, Bennett’s result for a Type-I specimen is 4 × 10^−8^ K^−1^ below the shaded area Wang et al. [8]; at 293.15 K, Bennett reports a difference in α(T) of 15 × 10^−8^ K^−1^ between Type-I and Type-III silica glasses.

Taken together, the preceding comments about the literature and Fig. 2(d) convey what motivated the present CTE measurements of fused quartz glass: neither measurements on the same glass recipe nor estimates of variability among recipes appear reliable at the ±10 % level in the temperature range of interest (273<T<303)K. Despite these staging remarks, the present measurement result for α(T) is no less surprising: the present work is clearly “on the low side,” and furthermore, the claimed uncertainty is 5.4 × 10^−10^ K^−1^, or 0.15 %. It is a struggle to coherently place the present “low” measurement in the context of historical reports. Regarding the Type-I fused quartz glass SRM 739, mutual consistency may only be claimed with the work of Drotning [15]; the present result is approximately 2.9u lower than Okaji and coworkers [16], and 2.7u lower than the foundational work of Hahn and Kirby [4]. [Here, u refers to the combined standard uncertainty of two measurements, and the quantity being compared is ∫α(T)dT for (263<T<313)K.] It is emphasized that the Type-I definition into which the tube material and SRM 739 are categorized is nominal, and it may be misleading to read too much into discrepant results. Moreover, Okaji and coworkers’ most recent measurements [8] cover five different recipes of fused quartz glass including Type I, compared to which they find α(T) of SRM 739 to be anomalously high. Notably, the present result is mutually consistent with all five recipes reported by Okaji and coworkers [8, 17], encompassing Type-I, II, and III silica glasses. Finally, it appears that the hitherto most accurate claim on a CTE measurement for a Type-I fused quartz glass was by Jacobs et al. [13]: the present measurements are only consistent with Ref. [13] above 295 K, if allowance is made for the 2 % to 3 % inhomogeneity observed in both cases.

A summary of the CTE measurements for all specimens in this work is presented in Table 1. The stated uncertainties on the fit parameters are statistical only and refer to the square root of the diagonal elements in the covariance matrix. The combined standard uncertainty for the measurement of α(T) is described in the next section. Acknowledging the observed inhomogeneity (imperfect reference material), these CTE measurements are among the most accurate to date. For this reason, and with the stipulation that the tubes have been laminated, the glass recipe and supplier [24] are specified [25]. The present measurements may serve as reference data for a specific blend and process of Type-I electric-fusion quartz glass. To this end, recommended values for the fit coefficients to be used with (2) are given in Table 1.

### 2.2 Measurement Uncertainty in α(T)

From (2), it is evident that u[α(T)] depends on only two things: how well one measures temperature and length. An uncertainty budget for the present measurement is listed in Table 2. Unless otherwise stated, all uncertainties in this work are one standard uncertainty, corresponding to a 68 % confidence level. The notation u(x) is used to denote the standard uncertainty of the quantity x. Before next describing each entry, it is pointed out that for simplicity all entries in Table 2 have been added in quadrature to produce the combined u[α(T)]. However, some entries have no temperature dependence and do not systematically affect α(T). Consequently, the uncertainty in ∫α(T)dT using Table 2 as written would be slightly overestimated. It is also emphasized that Table 2 only covers measurement uncertainty of a nominal fused quartz glass specimen, and includes no coverage for material inhomogeneity (see Table 1 and Ref. [13]).

The entry $‘‘L_{\text{ref}}’’$ in Table 2 refers to the length of the polished-ends tube spacer which forms the FP cavity. The optical length of an FP cavity may be determined within one part in 10^11^ by relating a measured resonance frequency to a mode number (and accounting for diffraction and mirror phase shifts). However, the length of the tube differs from the length of a FP cavity, and corrections are applied for mirror sagitta −49(10) μm, height of the mirror stack +8.0(1) μm, and penetration of the field into the coating −0.55(1) μm . A cosine error related to angular misalignment between the tube bore axis and the axis of the ${\text{TEM}}_{00}$ cavity mode introduces an additional 6 μm uncertainty. Overall, uncertainty in the tube spacer length $u(L_{\text{ref}})$ is a relatively small contributor when the FP cavity length is 333 mm. To be clear, $L_{\text{ref}}$ is the tube length at $T_{\text{ref}}=293.15\;K$, and the analysis of thermal expansion uses this reference value in $\alpha (T)=\frac{1}{L_{\text{ref}}}\frac{dL}{dT}$.

As mentioned, the FP-based approach to thermal expansion affords tremendous precision in tracking specimen length by inferring changes in cavity length dL via change in resonant frequency. The uncertainty in measuring a change in resonant frequency is 4 kHz (or 10^−11^ fractional), imposed by the stability of the iodine-stabilized HeNe laser reference. The entry “frequency” in Table 2 is negligible compared to other contributors. Rather than the ability to measure a resonant frequency, the measurement of dL is limited by imperfect materials and other mechanical or optical effects which disguise the temperature-induced length change of the spacer—the thing that is supposed to be measured. These other effects, unrelated to frequency metrology, are described next.

Temporal drift and instability in the cavities were, in fractional terms, 4.5 × 10^−11^ d^−1^ at 293.15 K. This estimate is based on a measurement of the change in resonant frequency while the cavities remained at vacuum for five days. Drift in cavity length may be corrected within 10 % across the 17 d of a measurement campaign, resulting in a 8 × 10^−11^ K^−1^ contribution to u[α(T)]. This stability assessment is of too short a duration to comment on longer-term effects, nor was there an obvious dependence of temporal stability on temperature, and these facts should be considered when making comparison to other work. That being said, the work of Berthold et al. [26] showed drift rates 5.6(3) × 10^−10^ d^−1^ and 5.1(3) × 10^−10^ d^−1^ for Type-II and Type-III silica glasses, respectively, over six months near 300 K. Excepting modern electronics, the present measurement technique is entirely analogous to Berthold et al. [26], but there is a factor 12 discrepancy in drift rates, which has no explanation. As stated above, the body of work by Jacobs and coworkers is very impressive—in Ref. [26] for example, they made great efforts to separate instability in optical contact from instability in the phase shift on reflection, and they also simultaneously measured cavities of different lengths—the work is impressive, but it is unclear how their apparatus [26] achieved frictionless support between the cavity and the chamber. A longer-term study with silica glass of unspecified type by Schödel and Abou-Zeid [27] employed a Twyman–Green large field imaging interferometer. Over seven years at 293.15 K, their sample exhibited a drift rate which slowed annually, ranging from 6.8 × 10^−11^ d^−1^ (initial) to 1.5 × 10^−11^ d^−1^ (final), with a fractional measurement uncertainty of about 4.9 × 10^−12^. A third study by Takahashi [28], lasted two years at 293.15 K, and monitored the change in separation between graduations of a line scale deposited on Type-III silica glass. The measurement technique employed a displacement interferometer and microscope, and estimated a fractional drift rate (0 ± 4.7) × 10^−11^ d^−1^. The observed short-term drift rates of the present work are consistent with the works of both Schödel and Abou-Zeid [27], and Takahashi [28]. (Note: Refs. [27, 28] both reported results on a yearly timescale, congruous with the sensitivity of their instruments—their reported data have been converted to a daily timescale.)

Continuing with the subject of cavity length instability, a different effect is now discussed: helium permeation into quartz glass. The present procedure soaked the cavities in 100 Pa of helium gas for 12 h to rapidly equilibrate the glass with the changing bath temperature. In this work, there was no evidence that the helium soak adversely affected the L(T) dataset, and an argument can be made, based on past experiences, why no instability was observed. Tests in Ref. [29] revealed that helium diffusion is described by one parameter that scales linearly with pressure. Near ambient conditions, the diffusion parameter takes a value approximately $2.5\times 10^{-9}\;h^{-\frac{1}{2}}$; the parameter describes fractional change in cavity length and at 100 Pa is projected to be 10^3^ times smaller than the high pressure work of Ref. [29]. The present work differs from Refs. [29] in one important respect: the previous work employed a cavity made with a binary glass of SiO_2_ and 8 % wt TiO_2_. On this score, Shelby [30] found no clear dependence on the (helium) permeation coefficient versus concentration of titania for a SiO_2_ binary glass (within a 14 % measurement error). Elsewhere, Avdiaj et al. [31] measured the permeation coefficient of a SiO_2_–TiO_2_ binary glass within an uncertainty of 8 %; Avdiaj et al. noted that their measurement matched the handbook value for a Type-III fused silica glass within 30 %. The preceding remarks offer good evidence that, for cavities made in fused quartz glass versus a SiO_2_–TiO_2_ binary glass, changes in cavity length caused by helium permeation would be the same within 20 %. Consequently, a reasonable prediction for cavity length instability can be based upon the older work [29] of SiO_2_–TiO_2_ binary glass with helium: one can predict that 12 h exposure to helium at 100 Pa gives rise to fractional instability in cavity length at the level of 8.7 × 10^−12^. This prediction is equivalent in magnitude to frequency instability in the iodine-stabilized laser or a 20 μK error in the measurement of glass temperature—at this level, helium soaking does not cause detectable changes in cavity length.

The effect of a potential mismatch in CTE between the mirror and the tube was modeled by the finite-element method. Two mismatch effects come into play [32]: mismatch between the spacer and substrate, and mismatch between the thin-film coating and the substrate. It was assumed that the mirror CTE was 5 × 10^−8^ K^−1^ larger than the tube CTE; this assumption covers the range of variability in CTE for the vitreous silica glasses shown in Fig. 2(d). It was assumed that the CTE of the thin film was 1.8 × 10^−6^ K^−1^; this assumption is an estimate of the mean value of silica and tantala [33, 34]. The model showed cavity length to anomalously increase by 40 pm·K^−1^, with 58 % arising from the thin film–substrate mismatch. Within this 40 pm·K^−1^ anomalous distortion, about 85 % of the effect was a “piston” displacement at the end of the cavity, and the remainder was mirror bending. That is, mirror bending was “outward,” increasing cavity length, and contributing a change to the radius of curvature (discussed more in the next paragraph). The present experiment with cavities of dissimilar lengths was purposely conceived to validate this model, but inhomogeneity in α(T) among tubes precluded any assessment; inhomogeneity in α(T) is 54 times larger than the effect of any likely mismatch in α(T) between mirror and tube. The estimate “mirror mismatch” in Table 2 is therefore model based, and refers to anomalous displacement at the ends of the specimen only. (Confidence in the finite-element estimates above was bolstered by cross-validation against the models of Fox [32] and Legero et al. [35]. It is emphasized that the estimates are specific to the present geometry: tube 18-mm outer diameter, inner diameter of tube bore at mirror contact 12 mm, mirror substrate diameter 25 mm, substrate thickness 10 mm, thin film coating diameter 8 mm, coating thickness 2 μm. As an example of the geometry dependence, mirror bending increases by a factor of 26 if the substrate were only 1 mm thick.)

Contributors arising from the simplified FP cavity resonance frequency are included in the entry “round-trip phase.” The complete expression for a resonance frequency $\nu =\frac{c}{2L}\left[m+\frac{\Phi (L)}{\pi }-\frac{\phi (\nu )}{\pi }\right]$ accounts for the Gouy phase shift and the phase shift on reflection. The Gouy phase $\Phi =\arcsin[{\left(L∕R\right)}^{1∕2}]$ depends on cavity length L and the mirror radius of curvature R; it arises from the difference in on-axis phase accumulated by a Gaussian beam versus a planar wavefront. The phase shift on reflection ϕ is given by the argument of the complex reflection coefficient from the dielectric mirror stack. The reflected phase depends on the properties of the stack (e.g., thin-film thickness and refractive index), and may be calculated by the transfer-matrix method [36]. Instead of $\frac{dL}{L}=-\frac{d\nu }{\nu }$, a more exact estimate for change in cavity length inferred by a change in resonance frequency

(4) $$\frac{\Delta L}{L}=\frac{\Delta \nu +\frac{c}{2L(T)}\left[\Delta m+\frac{\Phi (T)-\phi (T)}{\pi }\right]}{\nu },$$

takes into consideration a temperature dependence in both these round-trip phase terms—Φ and ϕ. For the Gouy phase shift, the temperature-induced cavity length change is approximately 0.4 (μm·m^−1^)·K^−1^, and therefore contributes $\frac{d}{dT}\Phi =0.28\;\mu \text{rad}\cdot K^{-1}$ to round-trip phase in the long cavity. Additionally, the finite-element model for a mismatch in tube-mirror CTE (previous paragraph) predicted an increase in sagitta $\frac{ds}{dT}=4\;\text{pm}\cdot K^{-1}$; assuming the concave portion of the mirror forms a chord of length l between the bonded annulus, the outward bending mirror contributes to a changing radius of curvature $\frac{dR}{dT}\approx -\frac{ds}{dT}\frac{l^{2}}{8s^{2}}$, or 0.3 μm·K^−1^ and $\frac{d}{dT}\Phi =0.64\;\mu \text{rad}\cdot K^{-1}$. In terms of resonant frequency, the net result on Gouy phase for temperature-induced changes in cavity length and radius of curvature is 100 Hz·K^−1^, which is a negligible effect. The temperature dependence of the phase-shift on reflection is more significant. A model of the mirror stack calculated [36] changes in ϕ (at fixed wavelength) as a function of temperature. The layers of the mirror stack were perturbed for the effects of the thermooptic $\frac{dn}{dT}$ dT and thermal expansion $\frac{dL}{dT}$ coefficients [33], and the model predicted $\frac{d}{dT}\phi =0.56\;\text{mrad}\cdot K^{-1}$ as the combined effect of two mirrors. The temperature dependence of ϕ(T) means that there is a systematic error of up to 80 kHz·K^−1^ in the dataset for the long cavity specimen, and 178 kHz·K^−1^ for the short specimens. Additionally, the group delay $\tau =\frac{1}{2\pi }\frac{d\phi }{d\nu }$ also exhibited a temperature dependence $\frac{d}{dT}\tau =0.2\;\text{as}\cdot K^{-1}$. The effect of $\frac{d}{dT}\tau$ causes a pseudorandom error in the estimate of ΔL, the magnitude of which depends on the value of the measured change in beat frequency Δf. In all datasets, 68 % of the Δf measurements fall within (180 ± 330) MHz, and since $\frac{d\nu }{dT}\approx 189\;\text{MHz}\cdot K^{-1}$ together with the average dT=3.1K in the procedure, one might expect random errors of ±231 pm·m^−1^ to appear in the datasets, uncorrelated between the cavities. In Fig. 2(b), there is no evidence of uncorrelated residuals this large, which suggests that the thin-film perturbations [33] to the mirror model are overestimated. Reference [33] states that thermal properties of thin films are not very well known—measurements are scarce, thin-film properties are believed to be considerably different than bulk material, and there is some ambiguity about the sign when there is a material property mismatch between the film and substrate. In any case, current knowledge of thin-film properties precludes any attempt at correcting for systematic or pseudorandom effects, and the entry in Table 2 expresses the inability to correct for ϕ(T).

The preceding paragraph revealed the fact that the change in resonant frequency has a $\text{modulo-}\frac{c}{2L}$ component. The present estimate of the cavity length L is based on a measurement of free spectral range $\Delta \nu_{\text{FSR}}=\frac{c}{2L}(1+\epsilon_{\tau })$, with $\epsilon_{\tau }=\frac{c\tau }{L}$ and $\tau =\frac{1}{2\pi }\frac{d\phi }{d\nu }$ being the group delay. In the present work, residual amplitude modulation in the laser systems was not canceled, and the present estimate of $\Delta \nu_{\text{FSR}}$ is no better than 1 kHz for the long cavity, or about 2 × 10^−5^ in fractional terms. Uncertainty in $\epsilon_{\tau }$ is twenty times smaller than this. The laser locked to the long cavity was tuned to a different resonant cavity mode number every 1.7 K on average. Consequently, error in the $\Delta \nu_{\text{FSR}}$-inferred value of cavity length causes a scale error of 17 kHz·K^−1^, but the entry in Table 2 for free spectral range shows it to be a small contributor overall.

Uncertainty in the temperature of the cavities is in sum the largest contributor to u[α(T)]. The master thermometer to which the thermistors were compared was a capsule-type standard platinum resistance thermometer which had been calibrated on ITS-90; the uncertainty in the ITS-90 fixed points is 0.1 mK. History on this particular cSPRT indicate that its calibration coefficients are no more stable than 0.5 mK over six months. The thermometer bridge used to measure the resistors has a specified nonlinearity in the measurement corresponding to 0.3 mK for this temperature-resistance ratio range. To correct for a temperature dependence in resistive heating (“self-heating”), all temperature measurements were referenced to zero power, but the present confidence in the correction is no better than 0.3 mK.

Finally, the last uncertainty contributor related to dT is a temperature gradient between the glass specimens and the thermometer. Two thermistors were used to measure gradients between opposite corners of the two aluminum cavity suspension enclosures shown in Fig. 1. Two thermopiles were used to measured gradients between the suspension enclosures and the isothermal flange (which housed the cSPRT) shown in Fig. 1. Between the redundancy of two thermistors and two thermopiles, gradients were detected within 0.3 mK. Nevertheless, the suspension enclosure is not the glass cavity, and this is relevant because the measurement technique is quasi-dynamic—100 Pa of helium was used to rapidly equilibrate the system during a temperature change, and pumping this gas out to measure the cavities at vacuum induced gradients up to 2 mK between the isothermal flange and (colder) suspension enclosure. The glass–aluminum system of Fig. 1 approaches full concentric enclosure, where radiative heat transfer [37] between the two surfaces would be written $Q=A_{1}\sigma \left(T_{1}^{4}-T_{2}^{4}\right)∕\left[\frac{1}{\epsilon_{1}}+\frac{A_{1}}{A_{2}}(\frac{1}{\epsilon_{2}}-1)\right]$, with the areas of glass and aluminum $A_{1}$ and $A_{2}$, temperatures $T_{1}$ and $T_{2}$, emissivities $\epsilon_{1}$ and $\epsilon_{2}$, respectively. When $T_{1}-T_{2}<10\;\text{mK}$, the series approximation $T_{1}^{4}-T_{2}^{4}=4(T_{1}-T_{2})T_{2}^{3}+\cdots$ is fractionally accurate within 10^−4^ near ambient. The energy balance between radiative Q and the cavity body rate of heat transfer $\rho Vc_{p}\frac{dT}{dt}$ then prescribes a thermal time constant $\tau =\frac{\rho Vc_{p}}{4A_{1}\sigma T_{2}^{3}}\left[\frac{1}{\epsilon_{1}}+\frac{A_{1}}{A_{2}}(\frac{1}{\epsilon_{2}}-1)\right]$, with glass density ρ, volume V, and specific heat $c_{p}$. This approximate model estimates τ=1.8h. Since there was a 12 h wait between helium pump-out and acquiring a L(T) data point, and since the system temperature is stable within 0.5 mK in that time frame, any few millikelvin gradients between the aluminum and glass created by pV work would be well under 0.5 mK after six time constants. Assigning an uncertainty of 0.5 mK to undetected gradients is therefore overcautious—especially since steady-state gradients measured between ends of the two aluminum enclosures were always less than (0.22 ± 0.15) mK—but this reflects the lack of comfort on this point: actual glass temperature was not measured, rather a low-emissivity body enclosing the glass, in vacuum, which had been cooled from pV work.

The bath and plumbing system employed was optimized for thermal performance, and openings in the thermal shells are few and small. This approach is at odds with good vacuum practice. For the CTE measurements, a 50 mm outer diameter vacuum hose was run into the bath, but the chamber was pumped through a 17 mm inner diameter flange. Ultimate vacuum inside the chamber was no better than 40(10) mPa. Steady-state residual gas is not an error, but pressure fluctuations change the refractive index between the cavity mirrors, and this change in optical length would be an error, if unaccounted for. Chamber pressure was continuously monitored with a diaphragm gage: measurement indicated residual pressure did not fluctuate by more than 10 mPa. The residual pressure was most likely water vapor, but small amounts of helium would also be present: not enough was known about its molar refraction to apply corrections to the resonance frequency. Assuming worst case of 10 mPa fluctuation of water vapor, CTE measurements in the long FP cavity would have errors of about 8 pm caused by changes in refractive index, corresponding to 2 × 10^−11^ K^−1^ in α, if measured across 1 K. The entry “residual gas” in Table 2 is therefore overestimated for the interest of scaling cell length over 20 K.

The entry “regression” in Table 2 is the quadrature sum of the root-mean-square error in the fit plus error in the fit model. Each of these components is now explained in turn. The residuals from the $\frac{L(T)}{L_{\text{ref}}}$ fit are plotted in Fig. 2(b). Note that these residuals exceed the present detection limit for a change in cavity length by more than an order of magnitude. The most likely explanation for the residuals, correlated in long and short cavities, is systematic error in measuring the cavity temperatures, on the order of 0.29 mK. As the previous entry for u(dT) shows, present confidence in thermometry is no better than 0.8 mK, and so the magnitude of the residuals is not surprising. The second (larger) component added in quadrature with the residuals is uncertainty in the fit model, and requires a brief preface. In literature there are different approaches to fit or deduce α(T). In the field of metrology, it has been customary to fit with polynomials [4, 8, 14, 18, 23]. Some recognize the correlation between thermal expansion and heat capacity via the Grüneisen parameter [38-40], and from there go on to recommend fitting based on an Einstein model with “empirical pseudo-quasi-harmonic [phonon] modes” [41-43]; or, when describing mid-range temperatures, functions with fewer free parameters [44]. Indeed, Reeber and Wang [42] explicitly advise against polynomials because they do not reliably extrapolate. Others take a hybrid approach, invoking an Einstein solid plus something else to describe low-temperature behavior—a quadratic term in the case of Swenson [45], and a Schottky-like function by Okaji [16]. The preceding remarks are but cursory: their purpose was to draw attention to the fact that there are several models to describe α(T), and thence with which to fit L(T). The choice of fit model becomes preponderant in high-accuracy work because as Martin et al. [46] point out, extracting α(T) is an ill-posed problem, since it is based on a derivative; consequently, the choice of model might lead to different answers for α(T). Martin et al. report a model-dependent deviation of up to 1 × 10^−9^ K^−1^ when deducing thermal expansion from measured L(T) data in a silicon specimen. In the present case, even in this limited temperature range, it was found that two pseudo-quasi-harmonic modes [42] are required to fit $\frac{L(T)}{L_{\text{ref}}}=b_{0}+\sum_{i=1}^{2}X_{i}\Theta_{i}∕[\text{exp}(\Theta_{i}∕T)-1]$, and describing

(5) $$\alpha (T)=\sum_{i=1}^{2}X_{i}{\left(\frac{\Theta_{i}}{T}\right)}^{2}\frac{\text{exp}(\Theta_{i}∕T)}{{\left[\text{exp}(\Theta_{i}∕T)-1\right]}^{2}},$$

requires four terms

$$X_{1}=-1.20(4)\times 10^{-6}\;K^{-1}\;\;\;\Theta_{1}=8.4(3)\;K\;\;X_{2}=2.14(4)\times 10^{-6}\;K^{-1}\;\;\;\Theta_{2}=549(6)\;K,$$

instead of the three terms of the quadratic in (2). It is thus unclear what benefit a pseudo-quasi-rigorous regression offers in this situation. [Insofar as the fit parameters of (5) are physically meaningful, $\Theta_{2}$ is related to the Debye temperature. Stephens [47] reported a Debye temperature of 494(25) K by measurement of the heat capacity of fused quartz glass. Okaji [16] inferred 535(9) K via measurement of CTE; their approach regressed α(T) and not L(T), and the first term of their fit function differed from (5).] In concurrence with the work of Martin et al. [46], it was found that (5) differs from the polynomial (2) by 1.2 × 10^−10^ K^−1^ standard deviation, which in fractional terms is α(T)—consistent with the $3\times 10^{-4}\cdot \alpha (T)$ finding of Ref. [46] for silicon. For the ultimate interest ∫α(T)dT, the difference between the models is less than 0.6 nm·m^−1^, which is a negligible concern. The entry “regression” in Table 2 is half the span of the fit model error added in quadrature with the root-mean-square error of the residuals from the fit.

To conclude, u[α(T)] listed in Table 2 has several contributors of similar magnitude, at the level of a few 10^−10^ K^−1^. Some of these have been incurred because the apparatus employs vacuum and thermal systems which were not designed for the purpose of CTE measurement. A different apparatus may be imagined, with greatly reduced chamber volume, increased vacuum conductance, and a thermal spray on all aluminum surfaces to increase emissivity. Such improvements might reduce u[α(T)] by about 40 %. However, breaking the 3 × 10^−10^ K^−1^ threshold appears challenging—it would require an understanding of thin-film coatings and reflected phase shift beyond state of the art.

### 2.3 Uncertainty Scaling $L_{\mathrm{CMM}}$ for Temperature

The previous subsection has discussed measurement uncertainty for a nominal fused quartz glass specimen. However, for the ultimate interest scaling $L_{\text{CMM}}$ for temperature, the uncertainty budget of Table 2 has almost no relevance—u[α(T)] is 24 times smaller than inhomogeneity among the tubes. Instead, uncertainty scaling $L_{\text{CMM}}$ for temperature is dominated by the information provided in Fig. 2(c) and Table 1, which are now discussed in more detail.

Figure 2(c) is an intercomparison of four specimens, with the specimen ${\text{FP}}_{333}^{\text{Type-}I}$ acting as the check standard. The additional specimen ${\text{FP}}_{154}^{\text{Type-}I}$ was briefly mentioned. Its creation was reactionary to the initial finding of large inhomogeneity. From Fig. 2(c) and recognizing $\Delta \alpha \approx \frac{1}{\nu }\frac{\Delta f_{\text{beat}}}{\Delta T}$ relative to the check standard ${\text{FP}}_{333}^{\text{Type-}I}$, the specimen ${\text{FP}}_{152}^{\text{Type-}I}$ exhibits a difference in α(T) of +1.1 × 10^−8^ K^−1^, and the specimen ${\text{FP}}_{154}^{\text{Type-}I}$ exhibits a difference of −0.2 × 10^−8^ K^−1^. So the range of inhomogeneity among three tube specimens is $\Delta \alpha =1.3\times 10^{-8}\;K^{-1}$. (Alternately, these estimates of Δα can be obtained in the final column $\frac{\Delta L}{L}$ of Table 1, recognizing ΔT=50K.) These tests of three specimens are not sufficient to make sound statistical inferences, but the work of Jacobs et al. [13] is again mentioned. They showed $\Delta \alpha =1.3\times 10^{-8}\;K^{-1}$ among thirteen specimens. Based on the three measured specimens in the present work, supported by the more thorough study of Jacobs et al. [13], it is felt that taking half the range $\Delta \alpha =1.3\times 10^{-8}\;K^{-1}$ is a reasonable decision. So, half the range of the three specimens measured is thought to cover inhomogeneity among all tubes used to make the gas cells, at the 68 % confidence level.

Next from Fig. 2(c) is that relative to the check standard ${\text{FP}}_{333}^{\text{Type-}I}$, the short cavity specimen increased by $\Delta \alpha =0.2\times 10^{-8}\;K^{-1}$ when it was modified from ${\text{FP}}_{152}^{\text{Type-}I}$ to ${\text{FP}}_{152}^{\text{potted}}$. This finding is not unexpected, because the potting compound has a generic mean CTE specification of 0.59 × 10^−6^ K^−1^. Covering uncertainty for the effect of potting compound, therefore, uses this measurement together with a length dependence, because the total “length” of compound used to pot the tube of ${\text{FP}}_{152}^{\text{potted}}$ was 60 mm, whereas the medium and long gas cells have 50 mm of their ends potted in compound. The measured change between ${\text{FP}}_{152}^{\text{Type-}I}$ and its potted ${\text{FP}}_{152}^{\text{potted}}$ gives a high level of confidence that the potting compound increases the effective thermal expansion of the gas cells. However, the somewhat uncontrolled nature of potting tubes (quantity used, layer thickness, squeeze out) means that a large uncertainty should be assigned to the effect. Specifically, for the 0.5 m cell which has 50 mm of each tube-end potted, the estimate is that the potting compound increases effective CTE by $7.0_{-0.0}^{+7.0}\times 10^{-10}\;K^{-1}$.

The final uncertainty estimate scaling $L_{\text{CMM}}$ for temperature is therefore the quadrature sum of CTE measurement uncertainty from Table 2, plus the effect of the potting compound, together with half the range of measured inhomogeneity; so that $u[\alpha_{\text{cell}}(T)]$ is 6.6 × 10^−9^ K^−1^, in which inhomogeneity dominates.

## 3 Conclusion

To conclude by clarification: this work does not advocate for fused quartz glass as a standard of thermal expansion—Fig. 2(d) is a strong case against such an endeavor. The thermal expansion measurements reported here are merely an attendant outcome of the quest to know $L_{\text{cell}}$ at 273.16 K, with the goal of establishing an optical pressure scale at the accuracy level of 1 μPa·Pa^−1^. The quest has been thoroughly justified: if cell length had been scaled for thermal expansion using the mean of Refs. [4, 15, 16], the consequence for the optical pressure scale would have been a bias error of at least 1.5 μPa·Pa^−1^, with an underestimated uncertainty for $u(L_{\text{cell}})$.

## Supplementary Material

supp-ijt-cte

## Figures and Tables

**Table 1:** Fit coefficients for (2) measured for each specimen, valid in the range $\left(263.15<T_{90}<313.15\right)$ K for *p* < 40mPa. Numbers in brackets are statistical uncertainty only. The recommended coefficients are the weighted average for all three Type-I specimens measured

Specimen	$a_{1}∕(10^{-7}\;K^{-1})$	$a_{2}∕(10^{-9}\;K^{-2})$	$a_{3}∕(10^{-11}\;K^{-3})$	$\frac{\Delta L}{L}=\int_{263\;K}^{313\;K}\alpha (T)\;dT∕(10^{-6})$
${\text{FP}}_{333}^{\text{Type-}I}$	3.95775(4)	3.0146(4)	− 1.299(3)	18.86
${\text{FP}}_{152}^{\text{Type-}I}$	4.06378(4)	3.0544(5)	− 1.306(3)	19.38
${\text{FP}}_{152}^{\text{potted}}$	4.0892(2)	3.044(3)	− 1.36(2)	19.50
${\text{FP}}_{154}^{\text{Type-}I}$	3.95122(5)	3.0567(5)	− 1.322(3)	18.81
Recommended	3.98263	3.0351	− 1.307	18.98

**Table 2:** Standard uncertainty in measurement of the coefficient of thermal expansion for a nominal-fused quartz glass specimen

Component	$u[\alpha (T)]∕(10^{-10}\;K^{-1})$
$L_{\text{ref}}$,12 μm	0.2
dL	
Frequency, 4 kHz	0.1
Instability, 15 pm·d^−1^	0.8
Mirror mismatch, 80 pm·K^−1^	2.4
Round-trip phase, 76 kHz·K^−1^	1.7
Free spectral range, 17 kHz·K^−1^	0.4
dT	
Calibration, 0.1 mK	0.5
Stability, 0.5 mK	2.5
Self-heat, 0.3 mK	1.5
Nonlinearity, 0.3 mK	1.5
Gradients, 0.5 mK	2.5
Residual gas, 10 mPa	0.2
Regression, 149 pm·m^−1^	1.5
Combined (*k* = 1)	5.4

No coverage for inhomogeneity [13] is included

## Data Availability

The supplementary material to this article is available on the NIST public data repository: https://doi.org/10.18434/mds2-2697. The supplementary material is an archive file, containing three sets of measurement data, which are: *Set 1: specimens ${\text{FP}}_{333}^{\text{Type-}I}$ and ${\text{FP}}_{152}^{\text{Type-}I}$ cycled in temperature side by side. *Set 2: specimens ${\text{FP}}_{333}^{\text{Type-}I}$ and ${\text{FP}}_{152}^{\text{potted}}$ cycled in temperature side by side. *Set 3: specimens ${\text{FP}}_{333}^{\text{Type-}I}$ and ${\text{FP}}_{154}^{\text{Type-}I}$ cycled in temperature side by side. *A Python script is included which reproduces Fig. 2, and also contains the historical reference data.

## Appendix

### Appendix 1: Enumerated Procedure

For clarity, here follows a step-by-step procedure on how the α(T) of the fused quartz glass was determined:

1. The FP cavity assembly of Fig. 1 was placed in a vacuum chamber.
2. The laser launched from the fiber optic collimators was aligned to the cavity axes. (Lower right part of Fig. 1.) The singlemode input fibers carried the laser from the ambient lab through the water bath and into the vacuum chamber.
3. The multimode output fibers were adjusted to receive laser transmitted through the cavities. (Upper left part of Fig. 1.) The multimode output fibers carried the transmitted laser out of the vacuum chamber through the water bath and out to the photodetectors at ambient conditions.
4. The water bath was filled with a 50:50 ethylene glycol and water mix. The outside of the vacuum chamber was submerged. The inside of the vacuum chamber was evacuated.
5. The setpoint of the water bath was adjusted, to 20 °C for example.
6. The vacuum chamber was filled with no more than 100 Pa of helium gas.
7. The vacuum chamber was left in 100 Pa for 10 h while the water bath setpoint was held constant.
8. The vacuum chamber was pumped to high-vacuum.
9. The lasers were locked to the respective cavities.
10. The readings of the two beat frequencies and the two thermistors were recorded over 12 h while the chamber remained pumped to high-vacuum. The two beat frequencies were (a) the beat frequency between the long cavity resonance and the iodine-stabilized laser frequency reference, and (b) the intercavity beat between the resonance frequencies of the long and short cavities. The beat frequencies were measured with frequency counters; the thermistors were measured with a resistance bridge.
11. The end of the 12 h data record became the final dL(T) datapoint, as given in the supplemental material. Change in specimen length dL was inferred by the measured change in resonance frequency Δν, via (4). The specimen temperature T was inferred by the measured temperature of the aluminum enclosure (see Fig. 1).
12. Steps 5 to 11 were repeated for the 17 set temperatures to acquire L(T)—i.e., specimen length as a function of temperature. During the acquisition of L(T), neither was the vacuum chamber opened, nor was realignment of the lasers to the cavity axes required.
13. The acquired L(T) was normalized as expansivity $\frac{L(T)}{L_{\text{ref}}}$ and fit with a cubic polynomial. See Fig. 2(a), and the implementation is given in the supplemental material.
14. The derivative of the function fitted to expansivity with respect to temperature yields the instantaneous coefficient of thermal expansion α(T). The function is given as (2), and is plotted in Fig. 2(d) of the main text.

Three sets of CTE measurements were performed, which compared different specimens. To change out a specimen (an FP cavity), the water bath was drained and the chamber brought to atmosphere and opened. The old specimen was removed and the new specimen was suspended from wires. All steps above were repeated.

The selection of the 17 set temperatures was based on instinct. A minimum requirement was that the three temperatures of interest were covered. The three temperatures of interest were the 20 °C operating point of the coordinate-measuring machine, and the two ITS-90 fixed points, water 0.01 °C and gallium 29.76 °C. Coverage of extrema by extending 10 °C was considered a good idea, and so was denser data around three temperatures of interest. The resultant (instinctive) set temperature selection is evident in Fig. 2(a). In general, the procedure began at 20 °C, was then set to −10 °C, then cycled incrementally in temperature up to 40 °C, and was finally repeated back at 20 °C. (Repetition back at 20 °C enabled correction of the small temporal drift.) Initial setup and characterization work showed no influence on L(T) from the direction of the temperature cycle.

## Appendix

### Appendix 2: Worked Examples of Uncertainty

The uncertainty analysis of the main text follows the usual approach of partial derivatives. The analysis operates on $\alpha (T)=\frac{1}{L}\frac{dL}{dT}$, and Table 2 is sectioned by the three main quantities involved. However, the α(T) is itself based on a derivative of fractional change in specimen length with respect to temperature. An additional complication is that some entries in Table 2 have no temperature dependence, but their effect on u[α(T)] manifests as statistical error on the determination of specimen length at each set temperature. Therefore, it is worthwhile to work through some examples from Table 2.

First, to take the largest uncertainty contributors: either “stability” or “gradients” from the u(dT) section of Table 2. These entries effectively mean that from set temperature to set temperature, the estimated dT has uncertainty. The origin of this uncertainty is because the temperature measured by the thermistor might not exactly represent the temperature of the specimen. The uncertainty contribution to u[α(T)] is $\frac{\partial \alpha }{\partial dT}u(dT)\equiv -\frac{1}{dT^{2}}\frac{dL}{L}u(dT)$. The $\frac{dL}{L}$ is nominally 0.5 × 10^−6^ K^−1^, and since u(dT)=0.5mK for a dT=1K change in set temperature, the entries in Table 2 are 2.5 × 10^−10^ K^−1^.

Next, the largest entry from the u(dL) section of Table 2 is “mirror mismatch.” This uncertainty arises because there might be a mismatch in material properties between the tube specimen and the mirrors that form the FP cavity. As temperature changes the mirror bends. Consequently, there is a difference between change in FP cavity length compared to the change in tube specimen length, which is the thing of interest. (Alternatively stated, the change in resonance frequency tracks change in tube specimen length plus mirror bending, rather than just change in specimen length alone.) As above, the uncertainty contribution to u[α(T)] is $\frac{\partial \alpha }{\partial dL}u(dL)\equiv \frac{1}{L}\frac{1}{dT}u(dL)$. The $u(dL)=80\;\text{pm}\cdot K^{-1}$ was estimated by finite-element analysis, which simulated for dT=1K. So, for the L=0.33m specimen, the entry in Table 2 is 2.4 × 10^−10^ K^−1^.

Finally, an entry like “regression” is estimated by fit statistics. Translating fit statistics to u[α(T)] might follow either the u(dT) or u(dL) path of the previous paragraphs; however, either path must chose something representative for dT. The estimate 149 pm·m^−1^ is based on root-mean-square residuals from the cubic fit to fractional length change across a temperature range of 50 K. The evidence from three independent measurements on the ${\text{FP}}_{333}^{\text{Type-}I}$ specimen suggested that regression error partially originates from calibration error in the thermistors. That is, the trend in Fig. 2(b) is smooth and reproducible as a function of temperature. Consequently, the appropriate interpretation is that the 149 pm·m^−1^ corresponds to u(dT)=0.29mK, and dT=1K is a representative interval for smooth variations. So, again u[α(T)] is $\frac{\partial \alpha }{\partial dT}u(dT)\equiv -\frac{1}{dT^{2}}\frac{dL}{L}u(dT)$. The $\frac{dL}{L}$ is nominally 0.5 × 10^−6^ K^−1^, and the entry in Table 2 is 1.5 × 10^−10^ K^−1^.
