# Supplementary material for: Expansivity of Fused Quartz Glass Measured Within 6 × 10−10 K−1
Source: Int J Thermophys. Author manuscript; Available in PMC 2024 Oct 4. (PMC11450646; doi:10.1007/s10765-024-03422-3)
Supplement: supp-ijt-cte [file NIHMS2022406-supplement-supp-ijt-cte.zip › apparatus-photo.pdf]

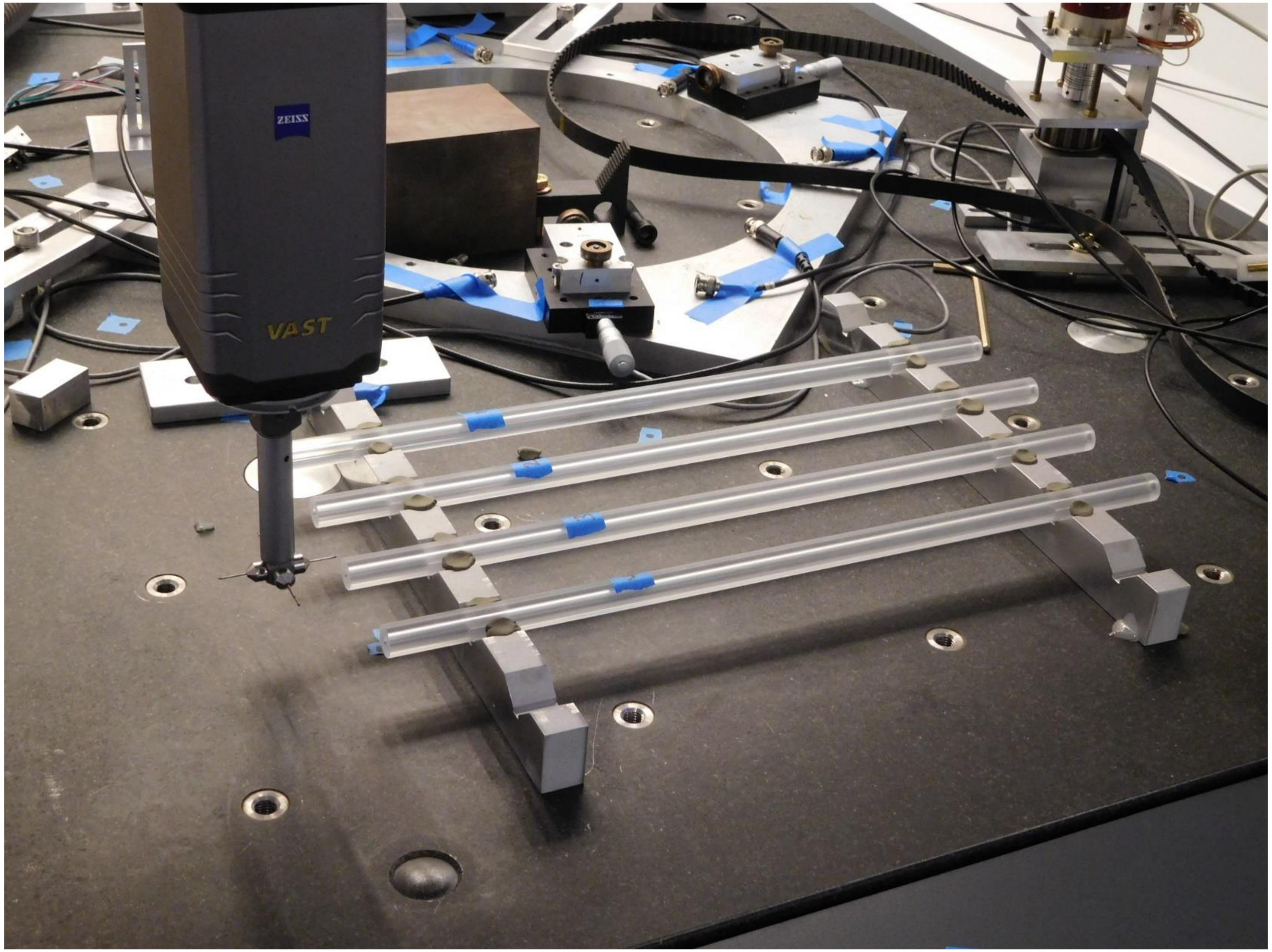

A bunch of fused quartz tube stock was purchased.

The end geometries were dimensionally characterized.

The tubes with most uniform end geometry were made into gas cells.

Some of the remainder tubing was cut up into CTE specimens.

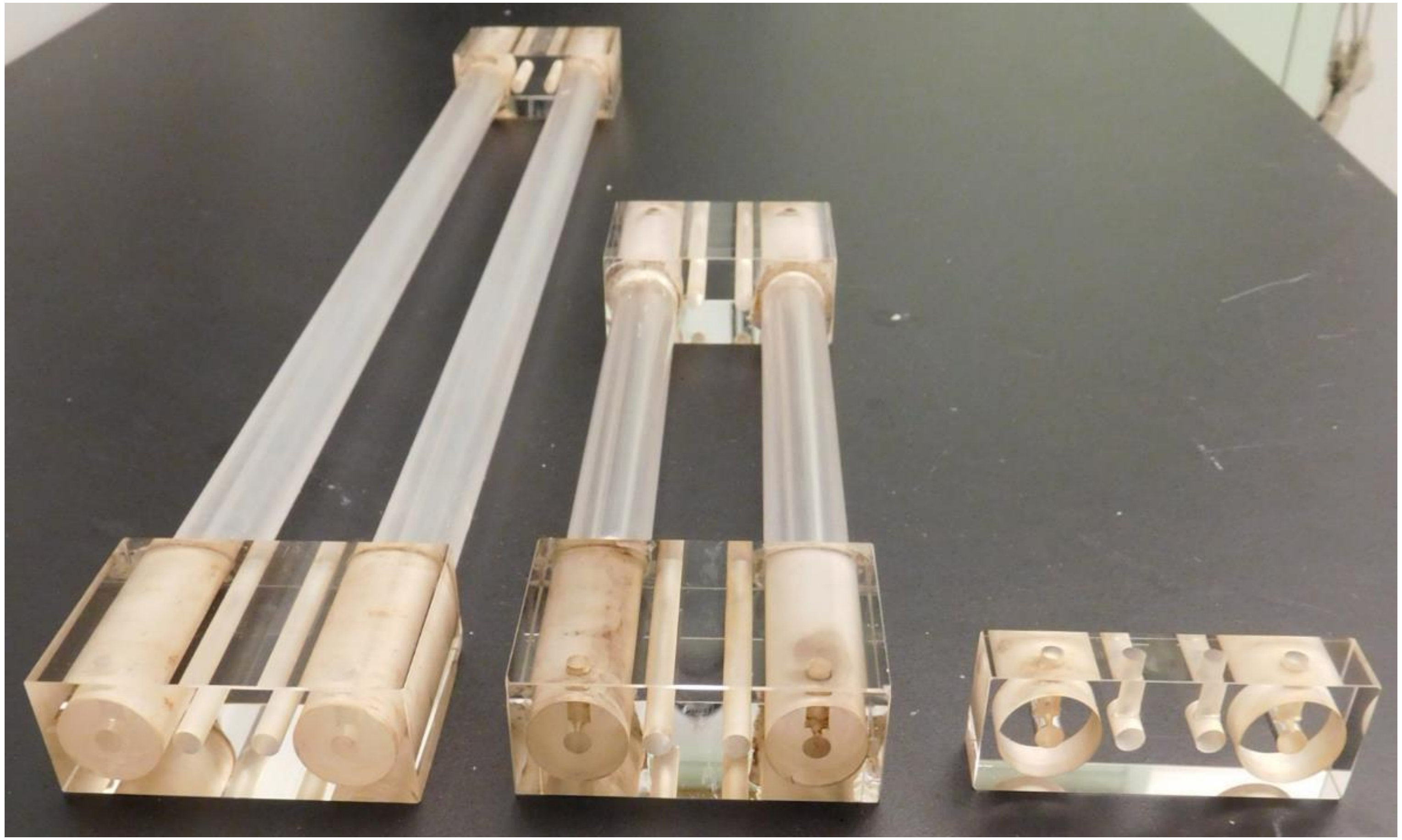

The three gas cells were manufactured by potting precision-bore fused quartz glass tubing into end blocks.

Cell lengths are 500 mm, 258 mm, and 15 mm. Cell lengths need to be dimensioned before the windows are bonded to the assembly.

This photograph is for background information only. These cells play no part in the determination of thermal expansion, reported in the main text.

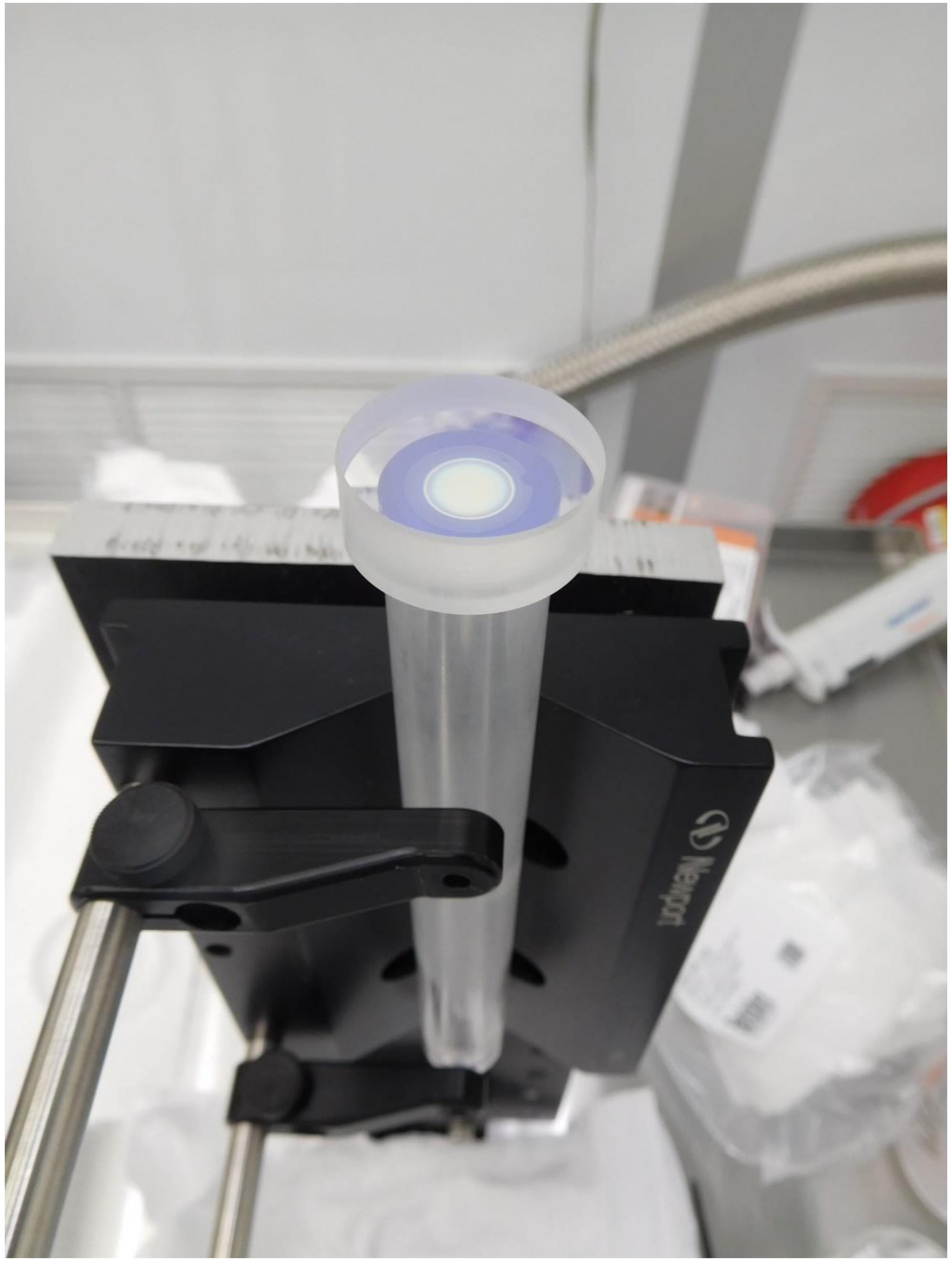

Some of the remainder tubing was cut up into CTE specimens

Each CTE specimen consisted of a tube with its ends polished, and with a pair of mirrors bonded on each end. This formed a Fabry-Perot cavity, which was configured plano-concave.

The mirror coatings were on fused silica substrates.

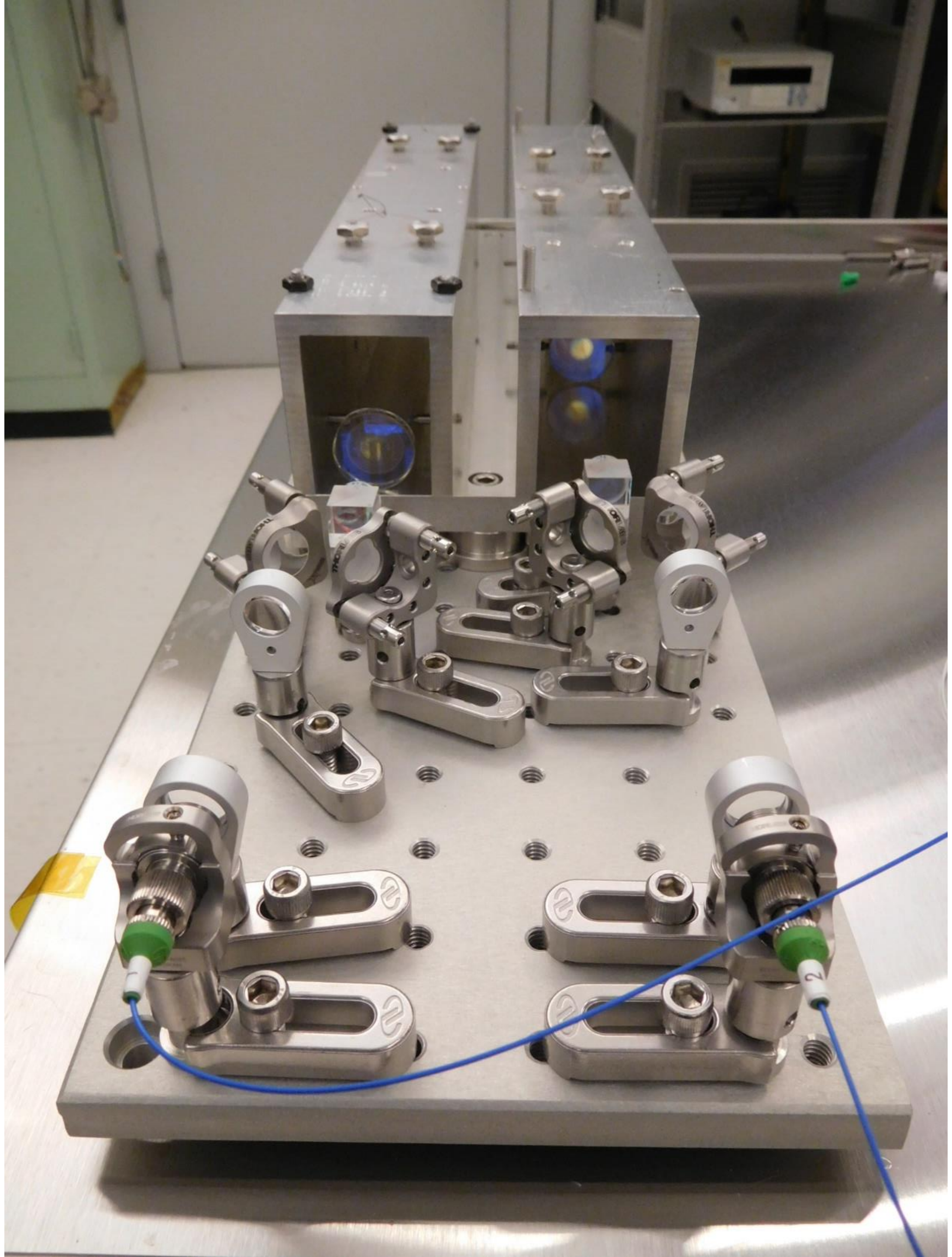

Two CTE specimens were compared side-by-side.

The specimens were suspended by wire, and friction-free to change length as a function of temperature.

All coupling optics were high-vacuum compatible.

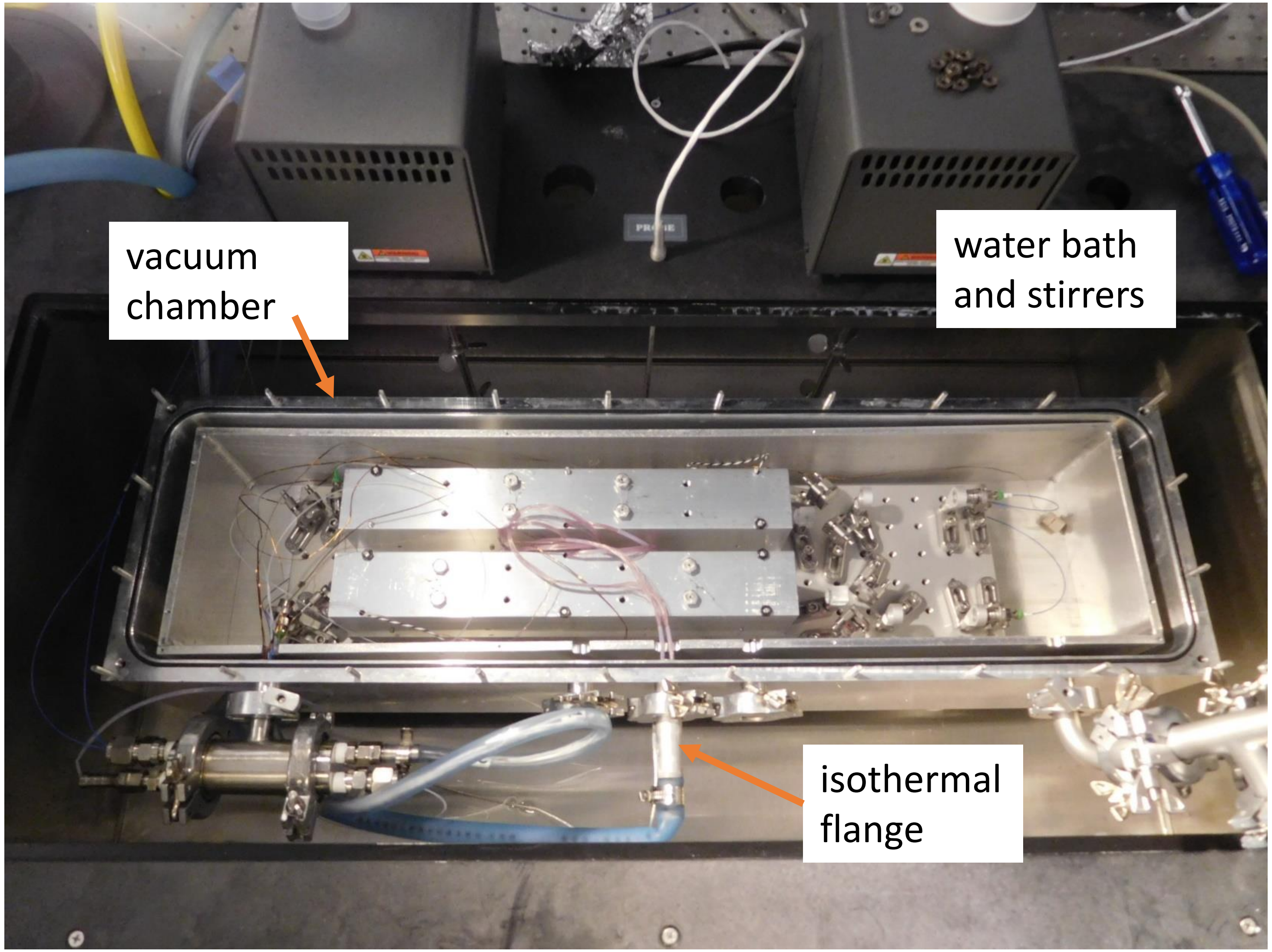

The entire optical assembly was placed inside a vacuum chamber with interior thermal shell, and submerged in a 150 L stirred waterbath.

Laser was fiber-in and fiber-out, through the water and vacuum interface.

Change in temperature was measured with thermistors, with redundancy from thermopiles

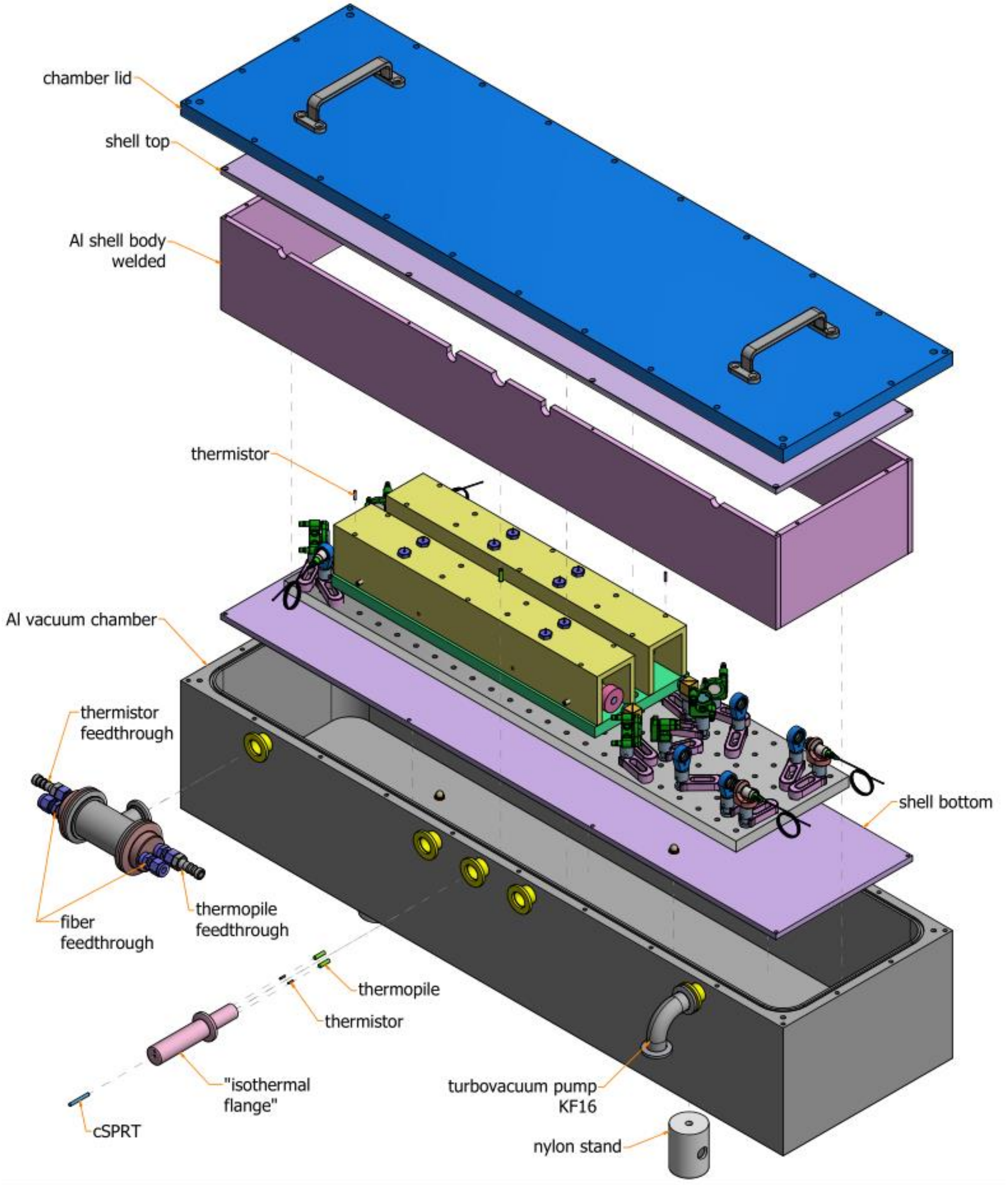

Exploded view of the apparatus, showing the FP cavity assembly and optics together with the vacuum chamber. This drawing corresponds to the photograph on the previous slide.

Note the “isothermal flange”, which functioned as a reference point. As described in the main text, gradients around the system were verified between thermistors and thermopiles, relative to this reference point.

The specimen temperature was inferred by the thermistors potted into the aluminum suspension enclosures (the yellow rectangles in the plot). The thermistors had been calibrated on ITS-90, relative to a capsule type standard platinum resistance thermometer.
